# Analysis of Motor Complication and Relative Factors in a Cohort of Chinese Patients with Parkinson's Disease

**DOI:** 10.1155/2020/8692509

**Published:** 2020-07-29

**Authors:** Baihua Sun, Tao Wang, Nianying Li, Jin Qiao

**Affiliations:** ^1^Department of Neurology, The First Affiliated Hospital of Xi'an Jiaotong University, Xi'an 710061, China; ^2^Department of Neurology, The Sengong Hospital, Xi'an 710300, China; ^3^Department of Rehabilitation Medicine, The First Affiliated Hospital of Xi'an Jiaotong University, Xi'an 710061, China

## Abstract

**Objective:**

Motor complications are common in Parkinson's disease (PD). The reported occurrence of motor complications varies across regions and races. The aim of our study was to describe the development of dyskinesias and motor fluctuations among Chinese PD patients and the relative risk factors.

**Methods:**

In the current cross-sectional survey study, PD patients with motor fluctuations and dyskinesia were enrolled from March to November 2018 in Shaanxi province, a northwest area of China. Data were collected by the movement disorder specialists. A self-designed questionnaire was utilized during face-to-face interviews. In addition, the relevant factors of motor complications were analyzed by univariable and multivariable analyses.

**Results:**

Of the166 PD patients recruited, 52 (31.33%) and 25 (15.06%) patients had motor fluctuations and dyskinesia, respectively, which occurred in 6.76 ± 3.77 and 8.61 ± 4.46 years after the onset of motor symptoms and 5.37 ± 3.33 and 6.80 ± 3.43 years after the treatment of levodopa therapy, respectively. Patients with motor fluctuations and dyskinesias had longer disease duration, younger onset age, higher Hoehn–Yahr stages and UPDRS III scores, higher daily levodopa dosage and levodopa equivalent daily dose (LEDD), and longer duration of levodopa treatment (*P* < 0.05). Bradykinesia-rigidity dominant patients had higher incidences of motor fluctuations (61.54% vs 38.46%) and dyskinesias (68.00% vs 32.00%) than tremor-dominant patients (*P* < 0.05). Results of the multivariate logistic regression analyses showed that the duration of levodopa therapy, age of the onset, and bradykinesia-rigidity dominant type were independent risk factors of motor fluctuations (*P* < 0.05). In addition, duration of disease and bradykinesia-rigidity dominant type were independent risk factors of dyskinesia (*P* < 0.05).

**Conclusions:**

The rate of motor fluctuations was higher than dyskinesias in Chinese patients with Parkinson's disease. Patients with younger age onset, bradykinesia-rigidity dominant type, longer disease duration, and longer duration of levodopa therapy are more likely to develop motor complications.

## 1. Introduction

Parkinson's disease (PD) is the second most prevalent neurodegenerative disorder of the central nervous system. Dopamine replacement therapy, which is considered as one of the main treatment methods, may lead to severe untoward effects, such as motor fluctuations and dyskinesias. Previous studies have indicated that approximately 40% PD patients who has been treated with levodopa for 4–6 years experienced wear-off (WO) and levodopa-induced dyskinesia [1], and 74% patients developed at least one motor complications within 4 years after starting treatment with levodopa [2, 3]. However, the incidence of motor complications is different in Asian countries. A Japanese investigation study showed that the percentage of patients with developed WO was 21.3%, 59.4%, and 73.2% by the end of the 5^th^, 10^th^, and 15^th^ year after disease onset, respectively [4]. In a Chinese multicenter registry survey, the incidence rates of WO and dyskinesia were 46.5% and 10.3%, which were lower than that in Western countries [5]. During the “on/off” periods, patients may experience fluctuations in motor and nonmotor symptoms. Another study reported that approximately 50% subjects may develop motor or nonmotor fluctuation within 5 years after levodopa therapy [6]. The etiology of motor complications is not well understood. Some studies have found that the patients with younger age of onset, longer duration of disease, higher daily levodopa dosage, severity of disease, and longer duration of levodopa therapy are more likely to develop motor complications. Few studies have shown that women, bradykinesia-dominant type, and low body weight are risk factors of dyskinesia [6]. Therefore, prevalence of motor complications and relative factors in PD is still controversial. In addition to the disease itself, treatment with levodopa is an important factor for the developing of motor fluctuations. New insight into motor complications of this disabled disease is urgently needed since they can be temporarily controlled but not fully eradicated. Therefore, it is crucial to clarify the potential risk factors for motor fluctuations and dyskinesia in different populations. The aim of the present study was to describe the development of dyskinesias and motor fluctuations among the PD patients, and related risk factors were also investigated.

## 2. Materials and Methods

### 2.1. Subjects and Basic Data

A cross-sectional study was performed on consecutive patients with PD. All patients were recruited from the First Affiliated Hospital of Xi'an Jiaotong University from March to November 2018 in Shaanxi province, a northwest area of China. All the participants were diagnosed according to Movement Disorders Society (MDS) criteria for Parkinson's disease [7]. Patients with severe cognitive dysfunction who could not complete study' rating scales were excluded. A self-designed questionnaire was used to ascertain the patients' information including gender, age, body massing index (BMI), disease duration, onset age, clinical types, and medications. Levodopa equivalent daily dose (LEDD) was calculated according to previous report [8].

### 2.2. Clinical Evaluation of Motor Complications and Clinic Characteristics

The presence of motor fluctuations included wearing-off (WO) and on-off phenomenon; WO was defined as “the generally predictable recurrence of motor and nonmotor symptoms before the scheduled doses of antiparkinsonian medication and improvements after next doses [9–11].” “On-off phenomenon” is characterized by ongoing predictable or unpredictable switching from being “on” with mobile with dyskinesia to being “off” and immobile during the course of a day [12]. Dyskinesia was defined as an involuntary, purposeless, predominantly choreic movement involving the muscles of the limbs, neck, trunk, or rarely face during the “on” period of PD patients [2]. Freezing of gait (FOG) is defined as “an episodic inability (lasting seconds) to generate effective stepping in the absence of any known cause other than parkinsonism or high-level gait disorders. It is most commonly experienced not only during turning and step initiation but also when faced with spatial constraint, stress, and distraction. Focused attention and external stimuli (cues) can overcome the episode [13]. ” Clinical assessments for motor fluctuations and dyskinesia phenomenon were performed in all patients. The severity of motor symptoms was recorded using the Unified Parkinson's Disease Scale part III (UPDRS III), and the PD disease severity was quantified using the modified Hoehn–Yahr stage. The tremor score was defined as the sum of UPDRS part II item 16 (self-report), part III item 20 (resting tremor), and part III item 21 (action/positional tremor). The nontremor score was defined as the sum of UPDRS part II items 5, 7, 12, and 15 and part III items 18, 19, 22, and 31. According to prevalent motor symptoms, subjects were divided into two subtypes: tremor predominant type (tremor score/nontremor score > 1) and bradykinesia-rigidity predominant type (tremor score/nontremor score ≤ 1).

All the enrolled patients agreed to participate in the study and gave full consent as dictated by the First Affiliated Hospital of Xi'an Jiaotong University ethics committee. All the assessments were performed by movement disorders specialists during face-to-face interviews with the patients.

### 2.3. Statistical Analysis

Statistical analysis was performed with SPSS version 24 (IBM). Normality of data for each individual parameter was assessed using the Schapiro–Wilk test. The differences in the clinical and demographic characteristics of the patients with and without motor complications and continuous variables, if normally distributed, were presented as mean ± SD, and two groups were compared by the 2-tailed *t*-test. If not normally distributed, continuous variables were presented as median (quartile) and compared by the nonparametric test. Discrete variables were compared by the chi-square test. A multivariate logistic regression analysis was performed. Factors with *P* < 0.1 were included in a multivariate logistic regression analysis to determine the independent risk factors for motor fluctuations and dyskinesia. *P* < 0.05 was considered statistically significant.

## 3. Results

### 3.1. Patients Characteristics

One hundred and sixty-six patients with PD including 89 men and 77 women, with a mean age of 65.80 ± 9.11 years, mean disease duration of 4.96 ± 3.90 years, and mean age of 60.83 ± 9.91 years at onset of disease, were enrolled in the study.  Table 1 summarizes their demographic data and clinical characteristics. According to the modified Hoehn–Yahr stage, 18 patients (10.84%) were in stage 1, 33 patients (19.88%) were in stage 1.5, 56 (33.73%) patients were in stage 2, 20 patients (12.05%) were in stage 2.5, 27 patients (16.27%) were in stage 3, and 12 patients (7.23%) were in stage 4. Eighty-nine patients (53.61%) were tremor-dominant types, and 77 patients (46.39%) were bradykinesia-rigidity types. The daily levodopa dosage was 468.87 ± 267.41 mg/d, and the duration of levodopa therapy was 2.93 ± 3.67 years.

Among them, 135 patients (81.33%) were treated with levodopa, 92 patients (55.42%) with dopamine agonists, 44 patients (26.51%) with monoamine oxidase B (MAO-B) inhibitor, 9 patients (5.42%) with catechol-oxyl-methyltransferase (COMT) inhibitor, 26 patients (15.66%) with anticholinergic drug, and 16 patients (9.64%) with amantadine.

### 3.2. The Characteristics and Prevalence Rates of PD Patients with Motor Fluctuations and Dyskinesia

Of all patients, 52 patients (31.33%) and 25 patients (15.06%) had symptoms of motor fluctuations and dyskinesia, respectively, which occurred 6.76 ± 3.77 and 8.61 ± 4.46 years after the onset of motor symptoms and 5.37 ± 3.33 and 6.80 ± 3.43 years after levodopa therapy, respectively. Additionally, 10 patients (6.02%) experienced freezing of gait.

Among patients with motor fluctuations, 46 patients (88.46%) experienced WO, and 6 patients (11.54%) experienced on-off phenomenon, Among patients with dyskinesia, 17 patients (68.0%) experienced peak-dose dyskinesia, 3 patients (12.0%) experienced biphasic dyskinesia, and 5 patients (20.0%) experienced dystonia.

### 3.3. The Relative Factors of PD Patients with Motor Fluctuations Based on Univariate Analysis

In comparison with patients without motor fluctuations, patients with motor fluctuations had older age, younger onset age, longer disease duration, more severely modified Hoehn–Yahr stage and UPDRS III; score, higher daily levodopa dosage and levodopa equivalent daily dose (LEDD), and longer duration of levodopa treatment (*P* < 0.05). In patients with motor fluctuations, the proportion of levodopa-benserazide, levodopa-carbidopa, dopaminergic agonists, COMT inhibitors, and amantadine was higher than that in patients without motor fluctuation (*P* < 0.05). Patients with bradykinesia-rigidity were more prone to motor fluctuations (*P* < 0.05). The specific results are shown in Table 2.

### 3.4. The Relative Factors of PD Patients with Dyskinesia Based on Univariate Analysis

In comparison with patients without motor complications, patients with motor dyskinesia had age older, longer disease duration, younger onset age, more severely modified Hoehn–Yahr stage and UPDRS III score, higher daily levodopa dosage and levodopa equivalent daily dose (LEDD), and longer duration of levodopa treatment (*P* < 0.05). In patients with dyskinesia, the proportion of levodopa-benserazide, levodopa-carbidopa, dopaminergic agonists, and COMT inhibitors was higher than that in patients with motor complications (*P* < 0.05). Patients with bradykinesia-rigidity were more prone to dyskinesia (*P* < 0.05). The specific results are shown in Table 3.

### 3.5. The Results of Motor Fluctuations and Dyskinesia in Different Clinical Stages

PD patients with younger age at onset tended to develop more motor fluctuations and dyskinesia, especially when under 50 years old. The incidence of motor fluctuations and dyskinesia were 62.07% and 31.03%, which occurred at 3.62 ± 4.29 and 2.54 ± 4.40 years of levodopa therapy. Motor complications increased with the duration of the disease and duration of L-dopa treatment. The dose of levodopa therapy was also related to the incidence of motor complication. PD patients taking higher daily doses, particularly over 600 mg/day, compared with lower doses of levodopa increased the risk of motor complications (*P* < 0.05). The specific results are presented in Table 4.

### 3.6. The Relative Factors of PD Patients with Dyskinesia Based on Multivariate Analysis

The results of multivariate logistic regression analyses showed that the duration of levodopa therapy, age of onset, and bradykinesia-rigidity dominant type were independent risk factors of motor fluctuations. In addition, duration of disease and bradykinesia-rigidity dominant type were independent risk factors of dyskinesia. The onset age was related to the risk of developing motor fluctuations (OR = 0.933, 95% CI: 0.888∼0.980); the younger onset was positive related with the risk of motor fluctuations. In addition, the clinical phenotype of PD was associated with the occurrence of motor complications. The occurrence of motor fluctuations in bradykinesia-rigidity dominant type was three times higher than that in tremor-dominant type (OR = 3.051, 95% CI: 1.164∼7.997), and the occurrence of dyskinesia in bradykinesia-rigidity dominant type was eight times higher than that in tremor-dominant (OR = 8.170, 95% CI: 1.862∼35.849). Patients with longer duration of levodopa therapy were more likely to develop WO (OR = 1.030, 95% CI:1.061∼1.043). The longer the duration of disease was, the greater the risk of dyskinesia was (OR = 1.359, 95% CI:1.161∼1.590). The specific results are shown in Table 5.

## 4. Discussion

Motor complications are frequent in patients with PD receiving dopaminergic replacement therapy. This cross-sectional study showed that 31.33% and 15.06% of Chinese PD patients experienced symptoms of motor fluctuations and dyskinesia. Compared with those reported in other populations, the prevalence of motor complications was relatively lower, especially dyskinesia. The reported rate of motor complications of PD patients showed a wide range, from 3% to 94%, mostly 30%–74% [1–5, 8]. Motor complications usually occurred after 4–6 years of levodopa treatment [2]. In a Japanese investigation of 1768 PD patients, the incidence of patients who developed wearing-off was 21.3%, 59.4%, and 73.2% at the end of the 5^th^, 10^th^, and 15^th^ year after disease onset [4]. In a Chinese multicenter registry survey (excluding patients in Northwest of China), the incidence of wearing-off and dyskinesia was 46.5% and 10.3%, respectively [5]. The results of this survey, which used the wearing-off questionnaire of nine symptoms (WOQ-9) to defined wearing-off, were similar to ours.

PD with early onset often develops dyskinesia, which may occur in the first two years of levodopa treatment, especially in patients who develop disease before age 50 [14]. In the present study, the prevalence of motor fluctuations in PD patients with disease onset at <50, 50–70, and after 70 years of age was 62.07%, 27.10%, and 16.67%, respectively. The prevalence of dyskinesia was 31.03%, 14.02%, and 3.33%, respectively. Ferguson et al. found that autopsy confirmed cases of early onset to PD (age at onset under 50 years) that had a higher cumulative incidence of dyskinesia, wearing-off, and on-off [15]. A 59-month follow-up cohort study showed that the younger age at onset was an independent risk factor of development of motor fluctuations [16]. These results suggested that it is appropriate to decide when to use levodopa therapy according to the age of onset.

In the present study, bradykinesia-rigidity dominant phenotype was an independent risk factor for motor fluctuation and dyskinesia. Previous observational studies showed that patient who presented tremor-dominant subtype as the initial manifestation was less likely to develop dyskinesia [17, 18]. This result was correlated with pathophysiological mechanisms of different clinical type. The tremor-dominant type is characterized by more severe cell impairment in the medial substantia nigra (SN) and more hyperactive in thalamo-motor and cerebellar projections. However, bradykinesia-rigidity dominant type, ventrolateral part of SN, and posterior putamen showed more severe cell loss, in which the glutamatergic thalamocortical pathway was inhibited, and cortical activation was reduced [19]. More recent studies showed that tremor-dominant PD patients had higher level of *α*-synucleinopathy and amyloid-*β* markers in cerebrospinal fluid than that in postural instability and gait difficulties phenotype [20].

Based on the logistic analysis, we found that the duration of levodopa treatment was significantly associated with the motor fluctuations. On the contrary, the duration of disease may be a risk factor of dyskinesia. Previous studies showed that the duration of levodopa treatment and disease duration were independent risk factors in generating wearing-off and dyskinesia [21, 22]. A study on Chinese PD patients found that duration of levodopa treatment was the best predictor of motor fluctuations, while disease duration was the best predictor for dyskinesias among the risk factors of motor complications [23]. The pathophysiology of wearing-off is multifactorial, involving both the loss of presynaptic dopamine neuronal and dopaminergic storage capacity, particularly in the anterior putamen and postsynaptic mechanisms. Dopamine receptor supersensitivity is thought to contribute to the development of dyskinesia. The buffering capacity of dopamine transporter is lost, and the short half-life of levodopa and pulsatile release of dopamine are considered to be the major risk factors for developing levodopa-induced motor complications. What's more, the overexpression and phosphorylation of glutamate system of N-methyl-D-aspartate (NMDA) receptors appear to be associated with the development of levodopa-induced dyskinesia [24]. Levodopa-induced dyskinesia has been found to be associated with genetic polimorphisms, such as the brain-derived neurotrophic factor (BDNF), DRD3G3127A, and DRD2/ANKK1 gene region [25, 26]. Some studies showed that levodopa may induce motor fluctuations and dyskinesia by dose-dependent manner. A study of sub-Sahara PD population comparing with matched Italian patients showed that motor fluctuations and dyskinesia were not associated with the duration of levodopa therapy, but with longer disease duration and higher levodopa daily dose [27].

A previous research has shown a significant and actual linear relationship between levodopa dose and dyskinesia [28]. The STRIDE-PD trial concluded that at the daily dosage of levodopa less than 400 mg may reduce the occurrence of motor complications [29]. A Chinese study showed that a safe levodopa daily dosage was not more than 275 mg or 4.2 mg/kg [21]. Cumulative levodopa dosage, which included both levodopa treatment duration and levodopa dosage, was another predictive of wearing-off [30]. In order to delay the onset of motor complications, some studies demonstrated that dopamine agonists in place of levodopa was first-line therapy [31]. In the present study, the main dopamine agonists used pramipexole and piribedil, and the proportions of these two dopamine agonists were higher in patients with motor complications compared to those without motor complications. Some studies have showed good efficacy of dopamine agonists in improving motor symptoms, and dopamine agonists can delay dyskinesia [32–34].

In conclusion, although levodopa remains the most effective treatment for PD and its use is related to better outcomes, for some patients with high risk of levodopa-induced motor complications, delaying the use of levodopa and reducing the dose of levodopa may be a better clinical choice. For patients with early stage, especially bradykinesia-rigidity type patients, dopamine agonists and MAO-B inhibitors may provide benefit when motor symptoms are not disabling and do not cause functional impairment in activities of daily life.

## Figures and Tables

**Table 1:** Demographic, clinical, and medical characteristics for our subjects.

Characteristics	Value
Gender (male/female)	89/77
Age (years, mean ± SD)	65.8 ± 9.11
Onset age (years, mean ± SD)	60.83 ± 9.91
Disease duration (years, mean ± SD)	4.96 ± 3.90
Clinical phenotypes (*n*, %)	
Tremor	89 (53.61)
Bradykinesia-rigidity	77 (46.39)
Modified Hoehn–Yahr stages (*n*, %)	
1	18 (10.84)
1.5	33 (19.88)
2	56 (33.73)
2.5	20 (12.05)
3	27 (16.27)
4	12 (7.23)
UPDRS scores (mean ± SD)	39.93 ± 18.80
UPDRS III scores (mean ± SD)	22.19 ± 11.83
Daily levodopa dosages (mg)	474.20 ± 268.76
LEDD (mg/d, mean ± SD)	457.48 ± 386.00
L-dopa duration (years, mean ± SD)	2.93 ± 3.68
Medications (*n*, %)	
Levodopa-benserazide	135 (81.33)
Levodopa-carbidopa	11 (6.63)
Dopamine agonist	92 (55.42)
MAO-B inhibitor	44 (26.51)
COMT inhibitor	9 (5.42)
Anticholinergic	26 (15.66)
Amantadine	16 (9.64)

Abbreviations: SD, standard deviation; UPDRS: unified Parkinson disease rating scale; LEDD: levodopa equivalent daily dose; MAO: monoamine oxidase; and COMT: catechol-o-methyltransferase.

**Table 2:** Characteristics of PD patients with or without motor fluctuations.

Motor fluctuations	With (*n* = 52)	Without (*n* = 114)	*t*/*χ*^2^	*P* value
Sex (male/female)	29/23	60/54	0.141	0.707
Age (mean ± SD, *y*)	63.55 ± 9.30	66.92 ± 8.86	2.215	0.029
Onset age (mean ± SD, *y*)	55.33 ± 9.81	63.41 ± 8.93	5.232	<0.001
Disease duration (median, quartile, *y*)	7 (5∼11)	3 (2∼5)	7.293	<0.001
Clinical phenotypes (*n*, %)			6.991	0.008
Tremor	20 (38.46)	69 (60.52)		
Bradykinesia-rigidity	32 (61.54)	45 (39.47)		
Modified Hoehn–Yahr stages (median, quartile)	2.5 (2∼3)	2 (1.5∼2.5)	4.770	<0.001
UPDRS III scores (mean ± SD)	25.71 ± 13.93	20.59 ± 10.43	2.358	0.021
Daily levodopa dosage (mg, median, quartile)	500 (375∼750)	375 (375∼425)	3.353	0.001
LEDD (mg/d, mean ± SD)	670.07 ± 411.89	356.06 ± 329.37	6.475	<0.001
L-dopa duration (mean ± SD, *y*)	6.06 ± 3.98	1.52 ± 2.46	6.825	<0.001
Medications (*n*, %)				
Levodopa-benserazide	50 (96.15)	85 (74.56)	10.963	0.001
Levodopa-carbidopa	8 (15.38)	3 (2.63)	9.387	0.002
Dopamine agonist	38 (73.08)	54 (47.37)	9.553	0.002
MAO-B inhibitor	17 (32.69)	27 (23.68)	1.488	0.223
COMT inhibitor	9 (17.31)	0 (0.00)	20.862	<0.001
Anticholinergic	7 (13.46)	19 (16.67)	0.278	0.598
Amantadine	9 (17.31)	7 (6.14)	5.113	0.024

Abbreviations: SD, standard deviation; UPDRS: unified Parkinson disease rating scale; LEDD: levodopa equivalent daily dose; Mao: monoamine oxidase; and COMT: catechol-o-methyltransferase.

**Table 3:** Characteristics of PD patients with or without dyskinesia.

Dyskinesia	With (*n* = 25)	Without (*n* = 141)	*t*/*χ*^2^	*P* value
Sex (male/female)	16/9	73/68	1.227	0.259
Age (mean ± SD, *y*)	65.92 ± 10.08	65.84 ± 8.97	0.042	0.966
Onset age (mean ± SD, *y*)	55.92 ± 11.18	61.74 ± 9.46	2.755	0.007
Disease duration (median, quartile, *y*)	9 (6∼13.5)	3 (2∼5)	6.113	<0.001
Clinical phenotypes (*n*, %)			5.529	0.019
Tremor	8 (32.00)	81 (57.45)		
Bradykinesia-rigidity	17 (68.00)	60 (42.55)		
Modified Hoehn–Yahr stages (median, quartile)	3 (2.75∼3.5)	2 (1.5∼2.5)	5.569	<0.001
UPDRS III scores (mean ± SD)	33.08 ± 15.18	20.26 ± 10.03	4.065	<0.001
Daily levodopa dosage (mg, median, quartile)	500 (375∼750)	375 (375∼500)	2.175	0.030
LEDD (mg/d, mean ± SD)	796.15 ± 544.42	395.22 ± 314.25	4.723	<0.001
L-dopa duration (mean ± SD, *y*)	7.40 ± 3.81	2.15 ± 3.04	5.481	<0.001
Medications (*n*%)				
Levodopa-benserazide	24 (96.00)	111 (78.72)	4.173	0.041
Levodopa-carbidopa	7 (28.00)	4 (2.84)	21.731	<0.001
Dopamine agonist	19 (76.00)	73 (51.77)	5.405	0.025
MAO-B inhibitor	9 (36.00)	35 (24.82)	1.365	0.243
COMT inhibitor	5 (20.00)	4 (2.84)	12.199	<0.001
Anticholinergic	6 (24.00)	24 (17.02)	0.698	0.403
Amantadine	2 (8.00)	10 (7.02)	0.026	0.087

Abbreviations: SD, standard deviation; UPDRS: unified Parkinson disease rating scale; LEDD: levodopa equivalent daily dose; Mao: monoamine oxidase; and COMT: catechol-o-methyltransferase.

**Table 4:** The results of motor fluctuations and dyskinesia in different clinical stages.

	Total (*n* = 166)	Motor fluctuations (*n* = 52)	*χ* ^2^	*P* value	Dyskinesia (*n* = 25)	*χ* ^2^	*P* value
Age of onset (*n*, %)			16.625	<0.001		9.101	0.011
≤50 years	29 (17.47)	18 (62.07)			9 (31.03)		
51–70 years	107 (64.46)	29 (27.10)			15 (14.02)		
≥70 years	30 (18.07)	5 (16.67)			1 (3.33)		

Duration of diseases			40.806	<0.001		48.692	<0.001
<5 years	91 (54.82)	11 (12.09)			2 (2.20)		
5–10 years	59 (35.54)	28 (47.46)			12 (20.34)		
≥10 years	16 (9.64)	13 (81.25)			11 (68.75)		

Duration of L-dopa treatment (*n*, %)			67.265	<0.001		46.087	<0.001
≤2 years	98 (59.04)	7 (7.12)			0 (0.00)		
2–5 years	36 (21.69)	21 (58.33)			11 (30.56)		
5–10 years	23 (13.86)	17 (73.91)			9 (39.13)		
≥10 years	9 (5.41)	7 (77.78)			5 (55.56)		

Modified Hoehn–Yahr stages (*n*, %)			29.030	<0.001		46.030	<0.001
1–1.5	51 (30.72)	6 (11.76)			0 (0.00)		
2–2.5	76 (45.78)	21 (27.63)			6 (7.89)		
3-4	39 (23.50)	25 (64.10)			19 (48.72)		

Daily levodopa dosages (*n*, %)			17.012	<0.001		6.880	0.032
≤300 mg	49 (29.52)	6 (12.24)			4 (8.16)		
300–600 mg	91 (54.82)	31 (34.07)			13 (14.29)		
≥600 mg	26 (15.66)	15 (57.69)			8 (30.77)		

**Table 5:** Results of logistic regression analysis in patients with motor fluctuations and dyskinesias.

		*B*	Walds, *χ*^2^	OR	95% CI	*P* value
Motor fluctuations	Duration of levodopa therapy	0.029	19.754	1.030	1.016∼1.043	<0.001
	Bradykinesia-rigidity type	1.115	5.417	3.051	1.164∼7.997	0.023
	Age of onset	−0.070	7.677	0.933	0.888∼0.980	0.006
	Constant	2.884	3.492	17.885		0.062

Dyskinesias	Duration of disease	0.307	14.653	1.359	1.161∼1.590	<0.001
	Bradykinesia-rigidity type	−2.100	7.750	8.170	1.862∼35.849	0.005
	Constant	−4.065	27.618	0.017		<0.001

Abbreviations: B: beta, OR: odds ratio, and CI: confidence interval.

## Data Availability

The data used to support the findings of this study are included within the article.
